# Catastrophic failure and metallosis of the acetabular component in total hip arthroplasty

**DOI:** 10.1186/s13018-021-02492-5

**Published:** 2021-05-29

**Authors:** Fırat Ozan, Murat Kahraman, Ali Baktır, Kürşat Gençer

**Affiliations:** 1 Department of Orthopedics and Traumatology, Kayseri City Hospital, Kayseri, Turkey; 2 Department of Orthopedics and Traumatology, Dünyam Hospital, Kayseri, Turkey

**Keywords:** Catastrophic failure, Metallosis, Wear, Bearing surface, Total hip arthroplasty

## Abstract

**Background:**

To evaluate the clinical features and results of the revision total hip arthroplasties (THA) in patients with catastrophic failures and metallosis.

**Methods:**

Fifteen hips of 14 patients with catastrophic failure and metallosis in hip arthroplasties were evaluated. They were followed for at least 4.2 years after the revision THA. Clinical evaluation was performed using Harris hip score. Osteolysis, loosening or presence of metallosis was evaluated with standard radiographs. Metallosis was evaluated intraoperatively according to the metallosis severity classification.

**Results:**

The mean time from failure until revision surgery was 9.4 years. It was observed that in the primary THA, metal-on-ceramic (MoC), ceramic-on-ceramic (CoC) and metal-on-conventional polyethylene (MoCPE) bearings were used in 1, 3 and 11 hips, respectively. Grade III metallosis was observed in all patients during revision surgeries. The mean Harris hip score increased from 55 points before revision THA to 75 points at the final follow-up. In revision arthroplasty, MoCPE and CoC bearings were used in 13 and 2 hips, respectively. The femoral stem was replaced in 5 hips. All acetabular cups, except that of one hip, were revised.

**Conclusions:**

Revisions of THAs with catastrophic failures and metallosis are quite challenging. Routine follow-up of arthroplasty patients is beneficial to examine for osteolysis, loosening, and asymmetric wear.

## Introduction

Total hip arthroplasty (THA) is an effective treatment for hip diseases [1, 2]. However, it can cause various complications, such as infection, periprosthetic fracture, destructive wear, particulate debris, osteolysis, metallosis, dislocation, heterotopic ossification, implant loosening and failure [1–6].

Catastrophic failure of THA components implies either their fracture or complete wear. It is a rare complication with an unclear aetiology [7, 8]. However, incorrect position of THA components, using the wrong material, poor material quality, failure of the locking mechanism of components, third body debris and increased patient activity are some of the probable causes [5, 9, 10]. Catastrophic failure of acetabular components can occur in 0.25–10.9% of THA patients [10]. It can be associated with different combinations of the bearing surfaces of the hip prostheses [10]. The reported incidence of the catastrophic failure of the femoral components ranges from 0.2 to 11% [8].

Metallosis is a rare complication that accounts for 5.3% of THA complications [6]. It is a component of catastrophic wear resulting from the debris released from the other arthroplasty components secondary to implant wear [6, 10, 11]. It is generally associated with metal-on-metal prosthetic devices, but it has also been described in non-metallic prostheses [6, 10].

Catastrophic component wear can continue to progress, generating many polyethylene (PE), ceramic and metal wear particles [6]. These wear particles are known to cause an inflammatory reaction and subsequent osteolysis, and their systemic absorption can cause clinical symptoms [3, 6, 9].

Wear of THA components may present with pain, instability, mechanical symptoms and squeaking [4, 5]. Additionally, the success rate of revision surgery performed after catastrophic failure is lower than that of other primary revisions [3, 12].

In this study, we evaluated the clinical features and revision arthroplasty results of patients who underwent THA with metallosis and catastrophic failure.

## Materials and methods

We evaluated 15 THAs in 14 patients (4 males, 10 females; 8 right hips, 7 left hips) that showed catastrophic failure and metallosis. Primary THA was performed at a mean age of 44.4 ± 9.7 years (22–60 years). The aetiology of primary THA was femoral neck fracture, osteonecrosis of the femoral head, developmental dysplasia of the hip and degenerative arthritis in 1, 4, 4 and 6 hips respectively.

All patients underwent clinical and radiological evaluations. The general complaints of patients before revision THA were severe hip pain, dysfunction, decreased mobility, leg length discrepancy and hip sounds. We evaluated metallosis intraoperatively according to the metallosis severity classification by Chang et al. [13] that defines 3 grades as follows: grade I (mild—black spotting in the soft tissues), grade II (moderate—geographically patterned black stain in soft tissues), and grade III (severe—black spotting throughout the soft tissues and bones).

Clinical evaluations were performed using Harris hip score [14]. Osteolysis, loosening or presence of metallosis was evaluated with standard radiographs. Osteolysis was defined as the presence of periprosthetic lytic lesions with a diameter exceeding 2 mm [3]. After revision THA surgery, in radiographic evaluation, a change in the angle of more than 4° or migration of more than 3 mm were taken as evidences of an unstable acetabular cup [3]. Instability of the femoral stem was defined as subsidence or vertical migration of more than 2 mm or a change in the stem angle of more than 2° on the anteroposterior hip radiographs [3].

## Results

**Fig. 1:**
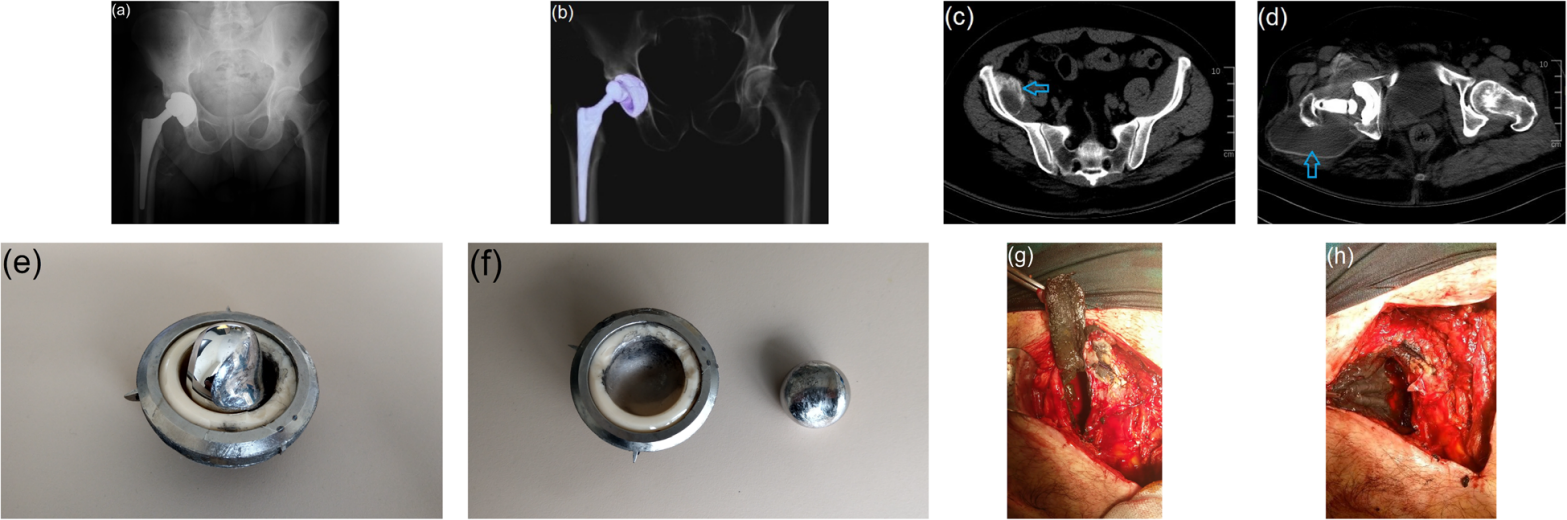
**a**, **b** Anteroposterior radiographic and 3D computed tomographic (CT) images of catastrophic acetabular component wear in a 49-year-old man with primary total hip arthroplasty with metal-on-ceramic bearing, 3 years postoperatively. **c**, **d** Axial CT images of the high-density pseudocystic structure extending into the pelvis in the right hip and surrounding the thigh widely (arrow). **e**, **f** View of deformation of the acetabular components. **g**, **h** Intraoperative appearance of diffuse metallosis developing in periprosthetic tissues

**Fig. 2:**
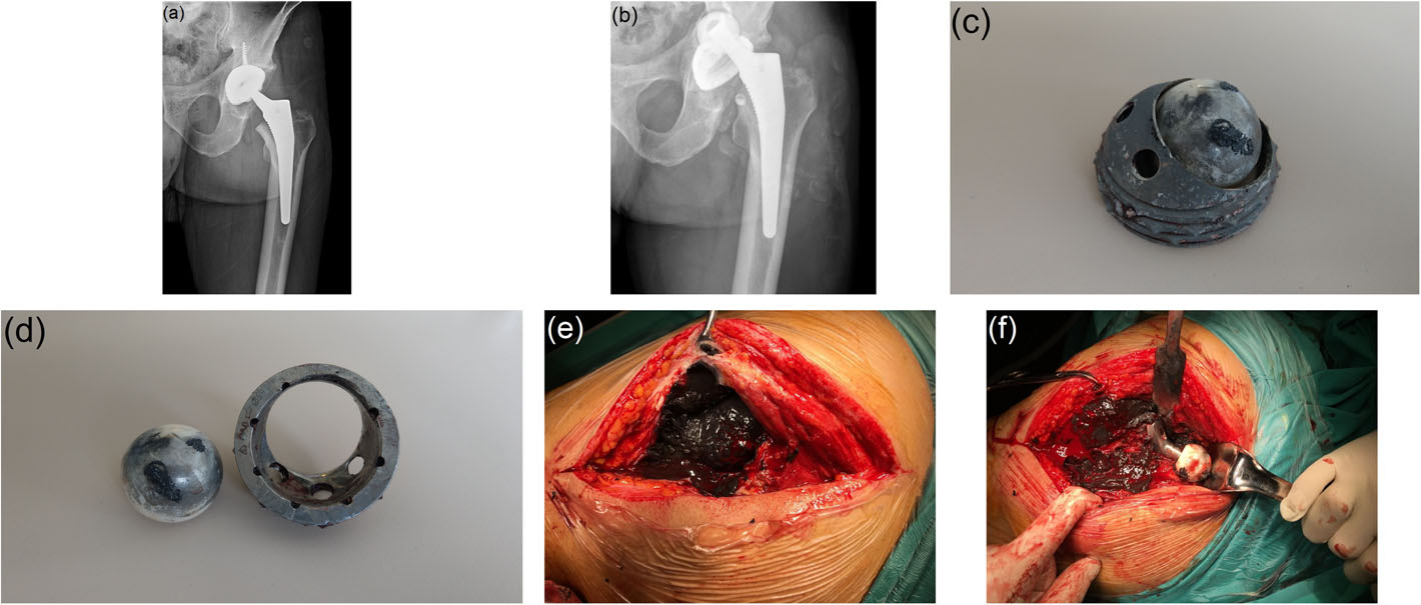
**a** Anterior-posterior (AP) radiographic image of a 60-year-old man with ceramic-on-ceramic bearing in the 1st year after primary total hip arthroplasty. **b** AP radiograph 4 years postoperatively showing penetration of the titanium acetabular cup of the ceramic head with complete wear of the ceramic liner. **c**, **d** View of the catastrophic deformation of acetabular components. **e**, **f** Intraoperative appearance of diffuse metallosis developing in periprosthetic tissues

**Fig. 3:**
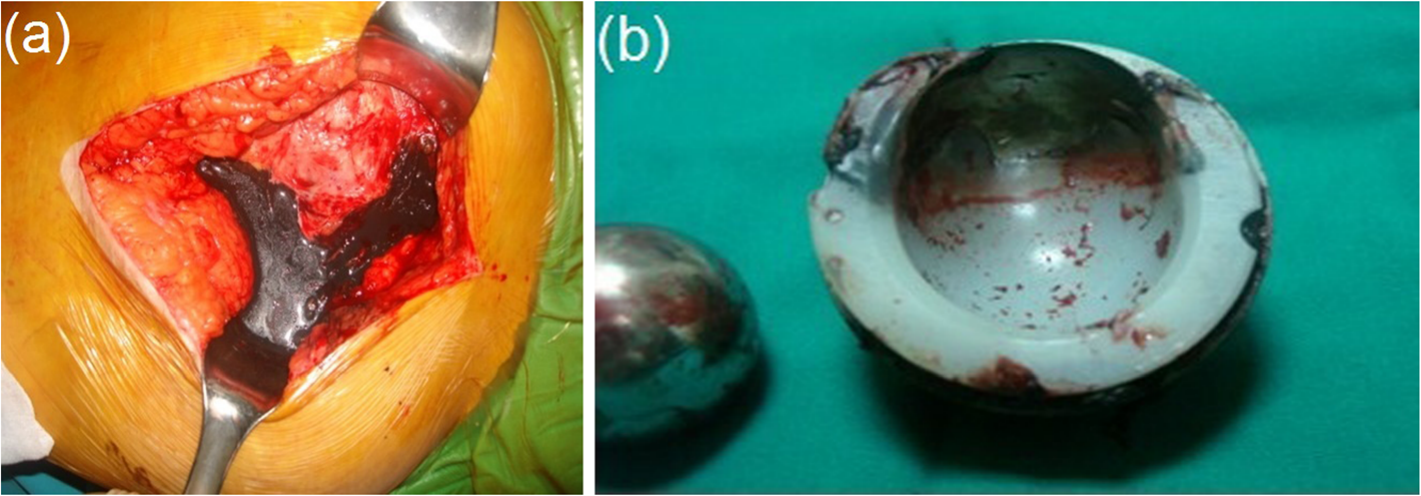
**a** Clinical picture of metallosis around the hip joint following catastrophic failure of polyethylene liner in a 41-year-old woman with primary total hip arthroplasty with metal-on-conventional polyethylene bearing, 4 years postoperatively. **b** The appearance of deformation in the polyethylene liner

The patient characteristics and clinical outcomes are presented in Table 1. It was observed that in the primary THA, metal-on-ceramic (MoC), ceramic-on-ceramic (CoC) and metal-on-conventional polyethylene (MoCPE) bearings were used in 1, 3 and 11 hips, respectively (Figs. 1, 2 and 3). We determined that cementless femoral stems were used uniformly, except in two hips. We further observed that in the primary THA, porous-coated titanium cups and cementless titanium expansion cups were used in 11 and 4 hips, respectively.

**Table 1:** Patient characteristics

	Sex	Age at primary THA, years	Time from catastrophic failure until revision surgery, years	Follow-up time after revision THA, years	Aetiology at the primary THA	Stem fixation at the primary THA	Primary THA bearing	Acetabular cup at the primary THA	Components of catastrophic failure	Stem fixation at the revision THA	Revision THA bearing	Acetabular cup at the revision THA
**1**	Female	54	7	7	Developmental dysplasia	Cementless	CoC	Porous coated titanium cup	Head, insert, cup	Not changed	MoCPE	Cemented titanium cup
**2**	Female	47	2	6	Developmental dysplasia	Cementless	MoCPE	Porous coated titanium cup	Head, insert, cup	Not changed	MoCPE	Cemented titanium cup
**3**	Female	46	6	8	Developmental dysplasia	Cementless	MoCPE	Porous coated titanium cup	Insert	Not changed	MoCPE	Not changed
**4**	Male	54	6	3	Fracture	Cementless	CoC	Porous coated titanium cup	Head, insert, cup	Uncemented femoral titanium stem	CoC	Porous coated titanium cup
**5**	Female	38	14	2	Osteonecrosis	Cementless	MoCPE	Porous coated titanium cup	Head, insert, cup	Not changed	MoCPE	Cemented titanium cup
**6**	Female	40	7	3	Degenerative arthritis	Cementless	MoCPE	Porous coated titanium cup	Head, insert, cup	Not changed	MoCPE	Porous coated titanium cup
**7**	Male	60	3	5	Degenerative arthritis	Cementless	CoC	Porous coated titanium cup	Head, insert, cup	Uncemented femoral titanium stem	MoCPE	Porous coated titanium cup
**8**	Male	49	3	4	Degenerative arthritis	Cementless	MoC	Porous coated titanium cup	Head, insert	Not changed	MoCPE	Porous coated titanium cup
**9**	Female	47	18	3	Degenerative arthritis	Cementless	MoCPE	Cementless titanium expansion cup	Head, insert, cup	Not changed	MoCPE	Porous coated titanium cup
**10**	Female	56	20	2	Degenerative arthritis	Cemented	MoCPE	Cementless titanium expansion cup	Head, insert, cup	Uncemented femoral titanium stem with modular neck	MoCPE	Porous coated titanium cup
**11**	Female	39	15	5	Osteonecrosis	Cementless	MoCPE	Cementless titanium expansion cup	Head, insert, cup	Not changed	MoCPE	Cemented titanium cup
**12**	Female	22	14	4	Osteonecrosis	Cementless	MoCPE	Cementless titanium expansion cup	Head, insert	Not changed	MoCPE	Porous coated titanium cup
**13**	Female	41	4	5	Developmental dysplasia	Cemented	MoCPE	Porous coated titanium cup	Head, insert, cup	Uncemented femoral titanium stem	MoCPE	Porous coated titanium cup
**14**	Male	37	10	5	Degenerative arthritis	Cementless	MoCPE	Porous coated titanium cup	Head, insert, cup	Uncemented femoral titanium stem	CoC	Porous coated titanium cup
**15**	Female	36	13	2	Osteonecrosis	Cementless	MoCPE	Porous coated titanium cup	Head, insert, cup	Not changed	MoCPE	Cemented titanium cup

The mean acetabular inclination angle was 53.6 ± 10.4° (range, 35–70°) in hips with primary THA. The femoral stem was detected at an average varus position of 10.5 ± 5.7° (range, 5–20°) in 4 hips.

All the revision THAs were performed due to catastrophic failure. The mean time from the failure until revision surgery was 9.4 ± 5.8 years (range, 3–20 years). The periprosthetic tissues appeared hyperdense, rounded and contoured on all hip radiographs. During the revision surgeries, we observed different signs of catastrophic wear on THA components, such as deformation in the femoral heads, fracture in ceramic inserts, excessive wearing of PE inserts and wear in acetabular cups. Grade III metallosis was observed in all patients during revision surgery.

In revision arthroplasty, MoCPE bearing was used in 13 hips and CoC bearing was used in 2 hips. The femoral stem was replaced in 5 hips and uncemented femoral stem was used. All acetabular cups, except those of one hip, were revised. Porous-coated titanium acetabular cups and cemented titanium acetabular cups were used in 9 and 5 hips, respectively. Bone grafts were placed in the acetabular defects in 3 hips.

The mean follow-up time of patients after revision THA was 4.2 ± 1.8 years (range, 2–8 years).

The mean Harris hip score increased from 55 points (range, 35–65 points) before revision THA to 75 points (range, 60–85 points) at the final follow-up. On radiologic evaluations after revision surgeries, there were no signs of osteolysis, loosening or instability during the follow-up period, nor were any other complications reported.

## Discussion

MoCPE arthroplasties have been performed safely for a long time [1, 2]. Additionally, revision rates of around 12% at 30 years with excellent pain and function scores have been consistently reported [1, 2]. However, increased life expectancy and higher patient demands have led to the evaluation of alternatives for the bearing surfaces in THAs [15]. Besides the standard MoCPE combinations, alternative bearing combinations, such as metal-on-highly-cross-linked polyethylene (MoXPE), metal-on-metal (MoM), CoC, ceramic-on-highly-cross-linked polyethylene (CoXPE) and ceramic-on-metal (CoM), have been used [15].

Conventionally, a minimum PE thickness of 6 mm throughout the liner dimension of MoCPE bearing is recommended [7, 10, 11]. If this thickness is less than 6 mm, the contact stress increases [7, 10, 11]. Also, femoral head roughness has been identified as source of exaggerated PE wear [7, 10, 11]. Other causes of catastrophic failure may include sizing mismatch, component malposition, third body wear and the PE processing and sterilization techniques [7, 10, 11]. Conversely, up to 40% of THA wear occurs due to unknown causes [7, 13].

In this study, varus malposition of the femoral stem was detected in 4 hips and acetabular component malposition was detected in 7 hips in the primary THA. However, we did not have direct evidence to prove that the failure occurred secondary to femoral head size mismatch, third body wear or PE processing and sterilization techniques.

Progressive wear of the liner leads to abnormal contact between the head component and the acetabular metal cup that causes unexpected friction between the two [6, 16]. Articulation of the cobalt-chrome or ceramic head with the softer titanium acetabulum leads to catastrophic failure [6, 16]. Metal wear particles are generated in addition to PE or ceramic wear particles, and finally, metallosis develops [3, 6, 9]. This condition is typically recognised by the accumulation of black periarticular soft tissues [4].

A substantial amount of liner wear debris and the infiltration of wear particles in the periprosthetic tissues lead to progressive bone loss and implant loosening after revision THA [3, 13]. Liner degradation has also been reported to result from backside corrosion such as shell-to-body or shell-screw, where there is direct impact between the said components [3, 15].

According to a case series, the probability of surviving THA was 85.7% at 15 years, 78.8% at 20 years and 77.6% at 25 years [1]. However, the survival rate of revision THA in patients with metallosis was found to be low [3, 13]. Kwak et al. [3] reported that the survival rate of revision THA in patients with metallosis following a catastrophic failure of a PE liner was extremely low. In their study, the survival rate after revision THA was 30.4%. Lee et al. [12] retrospectively reviewed the results of reoperation of 11 patients after their ceramic head and liner fractures were treated with MoP bearing replacement with a minimum follow-up period of 2 years. They reported that reoperations in such cases resulted in doubtful outcomes and concerns about metallosis. They suggested that reoperation with MoP bearing should not be performed for ceramic bearing fractures. A substantial amount of wear debris may lead to progressive bone loss and implant loosening, even after revision THA. In our study, we did not encounter any significant problems in revision THAs during the follow-up period. This may be due to our mid-term follow-up period or our aggressive tissue debridement.

Hard-on-hard bearings are one of the ways to reduce wear rates [2, 15, 17]. The reduced wear rates depend on the correct pairing of the bearing surfaces, surface roughness and roundness [2, 15, 17]. CoC bearing THAs fare better in terms of friction, lubrication and wear than MoXP, CoXP and MoM bearing THAs [2, 15, 17]. Furthermore, CoC prostheses may have less debris formation, osteolysis, loosening and prosthetic failures [15, 18, 19]. The average annual wear rate for CoC bearings is 0.1 mm^3^ for the femoral head and 0.04 mm^3^ for the acetabular liner [18, 19]. However, complications of CoC implants include hip squeaking and ceramic fracture [15, 17–19]. The latter is usually associated with pain and functional impairment of the affected joint [5]. Squeaking hip is a complication unique to THA having a hard femoral head in contact with a hard acetabular cup. However, squeaking hip is not always associated with pain or functional impairment [19].

Like CoC, MoM bearing surfaces are acceptable alternatives to classical MoPE bearing THAs due to their wear resistance and lower wear rates [15, 20]. Although MoM bearing THAs demonstrate lesser volumetric wear; however, the number of particles released is higher [20]. While the linear wear rates depend on many factors, their annual wear rates are in the range of 5–25% [15, 20].

The biological effects of wear particles and their corrosion products in the human body are largely unclear. However, it is known that the metal debris can induce pathological changes, such as the release of inflammatory cytokines from macrophages, histiocytosis, fibrosis and necrosis [20, 21]. Metal debris is thought to be associated with hypersensitivity and osteolysis [20, 22]. Inflammatory reactions may cause local and systemic alterations, depending on the metal type, particle size, volume and time of exposure [20, 22]. Local tissue reactions often need revision THAs. Revision THAs for adverse tissue reactions have been reported to have poorer outcomes when compared with those due to other causes [15, 23].

Increased incidence of malignancies in patients exposed to metal particles has not been reported [15, 17, 24]. However, in patients with MoM bearings, hypersensitivity to metals is higher than in normal population, especially in those with failed implants [15, 17, 24]. In this study, colorectal adenocarcinoma was detected 2 years after revision THA in a patient with CoC bearing surfaces.

Acetabular component malpositioning leading to failure with MoM has been previously reported [25]. Especially vertically inclined cup mechanics lead to edge loading with excessive anteversion [15, 25]. The other reported sources of wear debris include the head-neck junction with a CoCr head, modular necks and backside wear of modular metal cup liners, including CoM and MoM [15].

A pseudotumour was first described as a soft-tissue mass associated with the metal-on-metal resurfacing THA that did not show malignant or infective characteristics [15, 26]. Cytotoxic effect of metal particles and immunological reactions play a role in the development of pseudotumours [15, 26]. Histologically, these present with necrosis and heavy macrophage infiltration [3, 15, 26]. Symptoms such as pain, presence of a palpable swelling, skin changes, instability, deep vein thrombosis and nerve palsy may be present clinically [15, 26].

In this study, cloud sign and bubble sign, which are known to be related with metallosis, wear or fracture of the prosthesis liner, were observed in all hips on plain radiographs taken before the revision THAs [3, 6]. Also, pseudotumours were detected in 6 failed hips. The acetabular components of these hips were determined as MoC, CoC and MoCPE bearings in 1, 2 and 3 hips, respectively. The advantages of the CoM bearings include lower risk of squeaking and component breakage than in CoC bearings. CoM bearings have also been reported to produce lesser acetabular wear rate and metal debris than in MoM bearings [15, 27].

However, a randomised controlled study comparing CoM and MoM bearing surfaces showed an increase in mean serum Co and Cr levels in both groups and no significant differences in the increase in serum metal ion levels between the groups [27]. Wear of CoM bearings was reported to be primarily metal wear due to the superior hardness of the ceramic [4, 17, 18]. Similar to MoM, CoM bearings are associated with increased local or systemic metal ion levels and adverse tissue reactions [15, 20].

While there are many alternatives for THA bearing surfaces, each one of them has certain unique features [3, 17, 25]. The recommended combinations of arthroplasty component materials derive from the studies performed by the investigators and the clinical information reported in the literature [17]. Collaboration among disciplines through multidisciplinary teams facilitates the emergence of novel concepts [28]. Therefore, in particular, there needs to be interconnection between basic sciences and clinical sciences [28].

There is limited information available about the in vivo wear behaviour of MoC combination with only one case report about MoC in the literature [17]. The clinical account of one of our patients is only the second case in literature about MoC bearings. Valenti et al. [17] did not consider MoC as a suitable alternative for primary hip arthroplasty, and we too are in agreement with this. In our patient with MoC bearing, we detected massive metallosis in the surrounding tissues and a psuedocyst extending over a wide area. We debrided the metallosis area as best we could without harming the surrounding tissues, but we were not completely successful. This should be taken into account before considering the said alternative.

This study had several limitations. First, the number of patients was relatively small. Given the early diagnosis of wear, correct positioning of THA components, using the appropriate material, and selection of the correct bearing surfaces, catastrophic failures are now relatively rare. Second, although the data were collected prospectively, the study was retrospective in design. Third, the follow-up period of the study was mid-term. The long-term survival rate of the revision THA due to residual wear particles is currently uncertain. In the future, we can further explore the mechanisms of catastrophe and metallosis development by examining the different combinations of the bearing surfaces of the hip prostheses. Our next step will be to provide long-term results for the patients we are currently being followed up.

## Conclusion

Catastrophic failure and metallosis of THA components are rare occurrences and revisions in such cases are quite challenging. Routine follow-up of arthroplasty patients is beneficial to examine for osteolysis, loosening and asymmetric wear. As patients can also remain asymptomatic until catastrophic failure, regular follow-up detects possible failure of components and may reduce the morbidity associated with catastrophic failure and the resultant revision. Furthermore, correct surgical planning that can ensure thorough debridement of the residues in tissues due to metallosis is essential for good results.

## Data Availability

Not applicable.

## Abbreviations

THA: Total hip arthroplasty
PE: Polyethylene
MoC: Metal-on-ceramic
CoC: Ceramic-on-ceramic
MoCPE: Metal-on-conventional polyethylene
MoXPE: Metal-on-highly-cross-linked polyethylene
MoM: Metal-on-metal
CoXPE: Ceramic-on-highly-cross-linked polyethylene
CoM: Ceramic-on-metal
